# Lifestyle and socio-demographic factors associated with high-risk HPV infection in UK women

**DOI:** 10.1038/sj.bjc.6603822

**Published:** 2007-05-22

**Authors:** S C Cotton, L Sharp, R Seth, L F Masson, J Little, M E Cruickshank, K Neal, N Waugh

**Affiliations:** 1 Department of Public Health, University of Aberdeen, Polwarth Building, Foresterhill, Aberdeen, Scotland; 2 National Cancer Registry Ireland, Elm Court, Boreenmanna Road, Cork, Ireland; 3 Histopathology Department, Queen's Medical Centre, University Hospital NHS Trust, Nottingham, England; 4 Canada Research Chair in Human Genome Epidemiology, Department of Epidemiology and Community Medicine, University of Ottawa, Ottawa, Ontario, Canada; 5 Department of Obstetrics & Gynaecology, University of Aberdeen, Foresterhill, Aberdeen, Scotland; 6 Division of Epidemiology and Public Health, School of Community Health Sciences, University of Nottingham Medical School, Nottingham, England

**Keywords:** HPV infection, lifestyle factors, cervical cancer

## Abstract

The world age-standardised prevalence of high-risk HPV (hrHPV) infection among 5038 UK women aged 20–59 years, with a low-grade smear during 1999–2002, assessed for eligibility for TOMBOLA (Trial Of Management of Borderline and Other Low-grade Abnormal smears) was 34.2%. High-risk HPV prevalence decreased with increasing age, from 61% at ages 20–24 years to 14–15% in those over 50 years. The age-standardised prevalence was 15.1, 30.7 and 52.7%, respectively, in women with a current normal, borderline nuclear abnormalities (BNA) and mild smear. In overall multivariate analyses, tertiary education, previous pregnancy and childbirth were associated with reduced hrHPV infection risk. Risk of infection was increased in non-white women, women not married/cohabiting, hormonal contraceptives users and current smokers. In stratified analyses, current smear status and age remained associated with hrHPV infection. Data of this type are relevant to the debate on human papillomavirus (HPV) testing in screening and development of HPV vaccination programmes.

Infection with human papillomavirus (HPV) is necessary for the development of cervical cancer (Walboomers *et al*, 1999; Bosch *et al*, 2002). Around 40 HPV types infect mucosal surfaces of the lower genital area (International Agency for Research on Cancer, 2005) and are broadly classified into high- or low-risk for cervical cancer (Munoz *et al*, 2003). Testing for high-risk HPV (hrHPV) DNA has the potential to improve cervical screening (Brink *et al*, 2005). In addition, following encouraging trial results (Harper *et al*, 2004; Villa *et al*, 2005), two HPV vaccines are under licence. The effectiveness and cost-effectiveness of incorporating HPV testing into screening, and of vaccine programmes, will partly depend on current HPV prevalence, infection patterns and factors associated with infection within specific populations.

Human papillomavirus population prevalence mainly depends on patterns of sexual exchange (International Agency for Research on Cancer, 2005), which vary between and within countries, by, for example, birth cohort and ethnic group (Johnson *et al*, 2001; Fenton *et al*, 2005). Most available data on HPV prevalence and associated factors are from the United States of America, and/or focus on young women; many series are highly selective and may lack generalisibility. Other series did not examine lifestyle risk factors (e.g. Cuzick *et al*, 2003; Cuschieri *et al*, 2004; Moss *et al*, 2004; Hibbitts *et al*, 2006; Kitchener *et al*, 2006). Most infections in women under 30 are transient (Koutsky and Kiviat, 1999; Nobbenhuis *et al*, 2001; Woodman *et al*, 2001); infection risk factors, and/or their relative importance, may differ between young and older women. In addition, while cytological smear grade is strongly associated with HPV prevalence (Cuzick *et al*, 2003; Cuschieri *et al*, 2004), it is less clear whether the relative contribution of lifestyle risk factors differs by smear grade. We investigated factors associated with prevalence of hrHPV types in a large series of UK women and compared them in younger and older women and by cytological smear grade.

## MATERIALS AND METHODS

### Study population

Subjects were women assessed for eligibility for TOMBOLA (Trial Of Management of Borderline and Other Low-grade Abnormal smears), a randomised controlled trial (RCT) of alternative management policies and HPV triage (TOMBOLA Group, 2006).

Women aged 20–59 years, resident in Grampian, Tayside or Nottingham, with a low-grade smear (mild dyskaryosis or borderline nuclear abnormalities (BNA)) taken routinely in the UK national cervical screening programmes (CSPs) during 01/10/1999–31/10/2003, with no previous treatment for cervical lesions, were eligible for TOMBOLA. Recruitment was in two phases: 01/10/1999–12/03/2001 and 13/03/2001–31/10/2003. During phase one, eligible women had no abnormal smears in the previous 3 years; during phase two, they had up to one BNA smear in the previous 3 years. In phase one, women with a BNA smear were invited to a recruitment clinic approximately 6 months later for a follow-up smear and a swab for HPV testing. Women with a mild smear during both phases, or a BNA smear during phase two, were invited to a recruitment clinic approximately 2 months later and a swab for HPV testing was taken. The swab, an endocervical sample, was taken with a cytobrush; this was immersed in 2 ml of sterile phosphate-buffered saline containing 0.05% thiomersal.

A total of 52% of eligible women attended a recruitment clinic, of whom 95% (*n*=5514) consented to participate; 27 were subsequently excluded because the smear was inadequate. Five thousand and seventy-four (92%) of the remainder provided an HPV sample.

Ethical approval was obtained from the joint Research Ethics Committee of NHS Grampian and the University of Aberdeen, the Tayside Committee on Medical Research Ethics and the Nottingham Research Ethics Committee. Participants provided informed consent.

### HPV testing

Analysis was performed 1–4 weeks after swab collection in a single laboratory (Nottingham). Human and viral DNA were extracted using the Qiagen UK kit (Crawley, West Sussex, UK) (QIAamp® DNA Mini Kit) following optimisation of the manufacturer's protocol. Each batch included negative controls containing only elution buffer AE. Extracted DNA was amplified and quantitated by type-specific real-time polymerase chain reaction (PCR) for the housekeeping gene, human betaglobin. Samples without recordable DNA levels were considered inadequate (*n*=36; 0.7% of 5074). Adequate samples underwent HPV PCR using GP5+/6+ consensus primers, followed by enzyme immunoassay for detection of 14 high-risk types (16, 18, 31, 33, 35, 39, 45, 51, 52, 56, 58, 59, 66 and 68) (Jacobs *et al*, 1997). Women were considered hrHPV positive (hrHPV+ve) if their sample had an optical density (OD) three times greater than that of the batch-negative control, implying they carried at least one of the hrHPV strains. Other women were classified hrHPV negative (hrHPV−ve). Information was not available on individual HPV strains.

### Questionnaire

At recruitment, all women completed a questionnaire on ethnic group, marital status, tertiary education, employment, reproductive factors, smoking and physical activity.

### Statistical analysis

Women were classified by smear status, defined by their ‘current’ smear and any past BNA smear. For women with a BNA smear during recruitment phase one, this was considered the ‘past’ smear, while the ‘current’ smear was the recruitment smear (results ranged from normal to severe dyskaryosis). For other women, the ‘current’ smear was the smear that triggered the invitation to participate in TOMBOLA. Six smear status categories were defined: normal and one previous BNA; BNA and no previous abnormal; BNA and one previous BNA; mild and no previous abnormal; mild and one previous BNA; and moderate or severe dyskaryosis and one previous BNA.

Socio-demographic characteristics of women providing, and not providing, an HPV sample were compared using *χ*^2^-tests. High-risk HPV prevalence was age-standardised to the truncated (20–59 years) world standard population. Multivariate unconditional logistic regression models were constructed to assess factors associated with hrHPV+ve risk. Since age modified the relationship between smear status and hrHPV status, these factors were fitted by a single variable, which combined the six smear status categories with two age groups (20–29 and ⩾30 years). The global likelihood ratio test (LRT) was used to assess the impact of lifestyle and socio-demographic factors on age-and-smear status-adjusted risk estimates; factors with *P*<0.1 were retained in the final model. To explore sub-group heterogeneity, the modelling was repeated stratifying by age (20–29 years; ⩾30 years) and current smear (normal; BNA; mild). All multivariate models had adequate fit (by the Hosmer and Lemeshow test; Hosmer and Lemeshow, 1989).

## RESULTS

Younger women, white women and those with a single BNA or mild smear were less likely to provide an HPV sample than older women (*χ*_3_^2^=8.30, *P*=0.040), non-white ethnic groups (*χ*_1_^2^=8.19, *P*=0.004) or those with a previous abnormal smear (*χ*_5_^2^=100.85, *P*<0.001). Since two of three centres did not take swabs from menstruating women, the proportion providing a sample varied by centre (*χ*_2_^2^=294.53, *P*<0.001). It did not vary by tertiary education level (*χ*_1_^2^=0.34, *P*=0.559).

**Figure 1:**
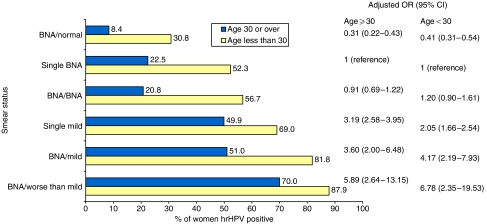
hr HPV positivity (%) by smear status and age. BNA, smear showing BNA; mild, smear showing mild dyskaryosis; worse than mild, smear showing moderate or severe dyskaryosis; single BNA, women with a current BNA smear and no other BNA smear in previous 3 years; single mild, women with a current mild smear and no BNA smear in previous 3 years. Adjusted OR: adjusted for age (in women aged 30 years or over – 30–34, 35–59, 40–44, 45–49, 50–54 and 55–59 years; in women aged less than 30 years – 20–24 and 25–29 years).

The crude hrHPV prevalence was 39.2% (95% confidence interval (CI) 37.8–40.5). Positivity declined with increasing age (20–24 years 61.0%, 25–29 years 50.1%, 30–34 years 39.6%, 35–39 years 30.6%, 40–44 years 22.0%, 45–49 years 17.1% and 50–59 years 14–15%), and increased with increasing smear grade. For all grades, prevalence and risk were higher in younger (20–29 years) than older (⩾30 years) women, but not by a constant amount (Figure 1; *P*(interaction) 0.0004). The overall age-standardised prevalence was 34.2% (95% CI 32.6–35.8). For women with a current normal smear, it was 15.1% (95% CI 12.6–17.6), for those with a current BNA smear 30.7% (95% CI 8.6–32.8) and with a current mild smear 52.7% (95% CI 48.6–56.8).

Other factors significantly associated with hrHPV status in multivariate analyses were tertiary education level, ethnic group, marital status, reproductive history, hormonal contraceptive use and smoking (Table 1). Women with a college/university degree were at reduced hrHPV+ve risk compared with those without a degree. Although prevalence varied little by ethnic group, in multivariate analyses, non-white women (e.g. black-African, Indian, Pakistani) were at significantly increased risk. Single, and divorced/separated/widowed, women had significantly higher infection risk than married/co-habiting women. High-risk HPV infection was associated with never being pregnant, having had children and age at first pregnancy, but not with number of children or caesarean delivery. Combining pregnancy, childbirth and age at first pregnancy (as ‘reproductive history’), having a pregnancy resulting in childbirth was associated with lower infection risk, particularly for a first pregnancy at age ⩾20 years. Current and previous oral contraceptive (OC) use (combined or progesterone-only), and current use of other hormonal contraception (e.g. implants, injections, intrauterine system), were associated with increased risk. Compared with never smokers, current smokers (but not ex-smokers) were at a modest increased risk, unrelated to smoking pack-years (data not shown). Barrier contraception and physical activity were also unrelated to risk.

In multivariate age-stratified analyses of women aged 20–29 years, age and smear status were significantly associated with infection. Tertiary education and having had children were also significant risk factors, with risk estimates similar to those in unstratified analyses. In women aged 30–59 years, age and smear status were significantly associated with infection, as were tertiary education, having had children, ethnicity and smoking; effect sizes were similar to those in Table 1. Other significant factors were marital status (increased risk in divorced/separated/widowed women (odds ratio (OR) 2.23, 95% CI 1.79–2.79) and single women (OR 1.84, 95% CI 1.35–2.50)), current hormonal contraceptive use (OR user *vs* non-user 1.30, 95% CI 1.03–1.64) and physical activity (OR active *vs* not active 0.76, 95% CI 0.60–0.97).

In multivariate smear-stratified analyses of all smear groups, age was significantly associated with infection. Having a college/university degree reduced infection risk in women with a current normal (OR 0.58, 95% CI 0.34–0.99) or BNA smear (OR 0.72, 95% CI 0.56–0.91) but not in those with a mild smear. In all smear strata, divorced/separated/widowed women had higher risk than married/co-habiting women (normal OR 1.64, 95% CI 0.91–2.96; BNA OR 2.26, 95% CI 1.73–2.95; mild OR 2.12, 95% CI 1.49–3.02). In the BNA strata only, being single also increased risk (OR 1.34, 95% CI 1.06–1.70). Having been pregnant was inversely associated with infection in those with a current normal (OR 0.60, 95% CI 0.38–0.95) or BNA smear (OR 0.81, 95% CI 0.64–1.02), but not in those with a mild smear. Having had children was associated with reduced risk in all smear strata, only reaching statistical significance in the current normal group (OR 0.57, 95% CI 0.36–0.92). Hormonal contraceptive use was associated with increased risk in the current normal (OR 1.59, 95% CI 1.02–2.48) and BNA (OR 1.29, 95% CI 1.05–1.59) strata, but not among the mild group. Barrier contraceptive use, caesarean delivery, smoking, physical activity and ethnicity were unrelated to infection in all strata.

## DISCUSSION

Our study was large, population-based and nested in a pragmatic RCT within the UK national CSPs. Among study participants, the current BNA : mild smear ratio (1.8 : 1) was close to that reported for the CSP screening age group in 2004–2005 (1.9 : 1) (NHS Health and Social Care Information Centre, 2005; ISD Scotland, 2007), suggesting our results are likely to be generalisable to women with low-grade smears.

While TOMBOLA participation was 52% overall, it was lower among younger women and those resident in more deprived areas (TOMBOLA Group, 2006), groups with increased HPV prevalence in this and other studies (Tonon *et al*, 1999; Cuzick *et al*, 2003; Winer and Koutsky, 2004). Thus, our crude hrHPV prevalence is likely to somewhat underestimate true prevalence among women with low-grade smears.

The treatment of lesions, and possibly also the act of taking a smear, can potentially clear cervical HPV infection (Shapiro *et al*, 2003; Sarian *et al*, 2005). Thus, hrHPV prevalence may be artificially lowered in populations with extensive screening coverage, such as the UK. Our participants had no previous treatment for cervical lesions and 66% had their last smear ⩾3 years before becoming eligible for TOMBOLA. Since hrHPV infection averages 8–14 months (Ho *et al*, 1998; Woodman *et al*, 2001), the effect of screening participation on our prevalence estimate is probably small.

A limitation of our study is that we did not collect information on numbers of sexual partners, age at first intercourse, etc, because of CSP guidelines (Duncan, 1997). Some factors we found to be associated with hrHPV infection may be markers of sexual behaviour. For example, smoking is associated with having had multiple sexual partners (Osler and Kjaer, 1996; Escobedo *et al*, 1997; Lam *et al*, 2001; Bellis *et al*, 2004; Jarvelaid, 2004), which is consistent with the observed raised infection risk among current smokers. UK rates of new partner acquisition vary by marital status, being highest among single or previously married women, intermediate among co-habiting women and lowest in married women (Johnson *et al*, 2001), a pattern compatible with our findings.

Our analyses extend existing knowledge on UK hrHPV prevalence, and are novel for Grampian and Tayside. Data from this and similar analyses will aid interpretation of studies of HPV testing in screening (e.g. Kitchener *et al*, 2006), and be valuable in modelling scenarios for alternative screening protocols, including HPV testing, as well as for policy makers in defining HPV vaccination strategies. It also provides a baseline against which the impact of vaccination on HPV infection patterns can be assessed in the future.

### Smear status

Other than age, current smear grade was the strongest predictor of infection. In women with a current BNA smear, our hrHPV prevalence (crude 34.2%, age-standardised 30.7%) was similar to that among women with a BNA smear from the UK ARTISTIC trial (31%) (Kitchener *et al*, 2006), but lower than (unstandardised) frequencies from other UK studies (46%; Moss *et al*, 2004, ∼55%; Hibbitts *et al*, 2006, 72%; Cuschieri *et al*, 2004), and for women with ASCUS (atypical cells of undetermined significance) smears from the US ALTS trial (49%; ALTS Group 2003). In the UK HART study, HPV prevalence among 289 women aged 30–60 years with a current BNA smear was 27% (Cuzick *et al*, 2003), close to the crude prevalence among women ⩾30 years in our study (26%). Our prevalence estimate among women with a current mild smear (crude 60.9%, age-standardised 52.7%) was also lower than non-standardised estimates from the United Kingdom and the United States of America of at least 70% (ALTS Group 2003; Moss *et al*, 2004; Hibbitts *et al*, 2006; Kitchener *et al*, 2006). In addition to different age profiles, comparison between studies is complicated by different HPV testing regimes (since tests detect different strains and vary in performance characteristics; Kulmala *et al*, 2004; Bosch and Iftner, 2005) and UK/USA differences in cytological abnormality classification.

Our crude (16.0%) and age-standardised (15.1%) prevalences among women with a current normal smear were similar to the pooled estimate from 27 PCR-based studies of cytologically normal women mainly from North America and Europe (16.2%) (Xi and Koutsky, 1997). However, our figures were higher than in recent UK screening studies with normal cytology reporting unstandardised frequencies of 6–10% (Cuzick *et al*, 2003; Cuschieri *et al*, 2004; Hibbitts *et al*, 2006; Kitchener *et al*, 2006). Our participants had had a previous BNA smear, which might inflate hrHPV prevalence, although among women with a current low-grade smear, having a previous BNA smear did not substantially increase prevalence (single BNA 34%; BNA/BNA 35%; single mild 60%; BNA/mild 69%). Our crude prevalence among women aged 30–59 years (8.4%) was similar to that from the HART study (6%) (Cuzick *et al*, 2003). Our age-standardised prevalence was similar to that for sexually active women aged 15–74 years from four countries in Latin America (12.4–17.7%) and parts of Asia (China, Korea, India; 14.0–16.8%), higher than for Vietnam and Thailand (1.6–11.4%) and lower than for Nigeria (27.0%) (Franceschi *et al*, 2006); these figures relate to 27 HPV types, including 6 and 11.

### Age

Notable geographical variations in age-specific HPV curves have recently been described (Franceschi *et al*, 2006). Our observation of decreasing hrHPV infection with increasing age (in all smear strata) is consistent with other UK, European and US series (Cuzick *et al*, 2003; Winer and Koutsky, 2004; Franceschi *et al*, 2006; Hibbitts *et al*, 2006).

### Ethnic group

In the United Kingdom, white, black-African and black-Caribbean women have higher numbers of lifetime sexual partners, lower median age at first heterosexual intercourse and higher incidence of (non-HPV) sexually transmitted infections than women from Indian or Pakistani ethnic groups (Fenton *et al*, 2005). Although we observed increased infection risk among non-white ethnic groups, relatively few women described themselves thus (*n*=223), precluding multivariate analysis of individual groups. Crude infection frequencies (Asian origin 35%; white 39%; black 45%) are consistent with sexual behaviour data and suggest that the raised risk may be limited to black women.

### Contraception

High-risk HPV infection risk was >50% higher in women who had used OCs or other hormonal contraceptives. The latter have been studied little previously. Among ALTS participants, no association was found with injectable contraceptives or Norplant (Castle *et al*, 2005). Previous studies of OC use have been inconsistent (Green *et al*, 2003; Winer and Koutsky, 2004; Vaccarella *et al*, 2006a), perhaps due to differences in study design, types of OCs used/assessed, prevalence of use and adjustment factors. While OC use may simply be a marker for ‘high-risk’ sexual behaviours (Winer and Koutsky, 2004), in several studies the OC–hrHPV association persisted after adjustment for factors such as number of sexual partners (Ley *et al*, 1991; Sikstrom *et al*, 1995; Winer *et al*, 2003). In further analyses, we found a stronger relationship between current, than past, OC use and hrHPV positivity (OR ex-users *vs* never users 1.23 (95% CI 0.99–1.51); OR current users *vs* never users 1.46 (95% CI 1.24–1.73)). This appears compatible with the postulated mechanisms by which current OC use influences infection, which include the enhancing effect of oestrogens on cervical ectopy, thereby permitting potential carcinogens (including HPV) easier access to the transformation zone (Green *et al*, 2003), and increased cell proliferation and transcription of HPV induced by direct hormonal effects on cervical cells (de Villiers, 2003).

### Reproductive history

Our observation that having been pregnant was associated with reduced hrHPV infection risk is consistent with the IARC HPV Prevalence Surveys Study Group analysis of >15 000 women (Vaccarella *et al*, 2006a). We found that the effect was stronger if a childbirth had resulted, and with older age at first pregnancy. In the United Kingdom, earlier age at first pregnancy or childbirth, and decreased likelihood of having an abortion, are associated with low socio-economic status (Smith, 1993; Wellings *et al*, 2001; ISD Scotland, 2006). However, our analysis was adjusted for tertiary education as a measure of socio-economic status. Although age at first pregnancy may be a marker for age at sexual debut, it is not as strongly predictive of HPV infection as previously thought (Vaccarella *et al*, 2006b). Possible explanations for this inverse association with pregnancy include breastfeeding, which results in high progesterone levels with atrophic changes and retraction of the squamocolumnar junction into the cervical canal, possibly reducing the likelihood of infection; alterations in patterns of sexual (e.g. changing partners, frequency of coitus) and other behaviours that influence infection risk (e.g. smoking).

### Age- and smear-stratified analyses

Identifying differences in the relative importance of risk factors in sub-groups can be informative – as is evident from the few previous studies using this analytical approach (Lazcano-Ponce *et al*, 2001; Molano *et al*, 2002; Anh *et al*, 2003). We undertook age- and smear-stratified analyses because these were the most important risk factors, and they interacted, suggesting that the relative contribution of hrHPV infection, and other factors, in the aetiology of cytological abnormalities differs by age.

## Figures and Tables

**Table 1:** Numbers and proportions of women hrHPV+ve and adjusted multivariate ORs for socio-demographic and lifestyle factors

	**Total (*n*)**	**hr HPV+ve (*n*)**	**% hrHPV+ve**	**Multivariate OR^a^**	**95% CI**
Overall	5038	1973	39.2		
					
*Tertiary education/training*					
No degree	4122	1656	40.2	1	Reference
Degree	887	305	34.4	0.72	0.61–0.87
Missing	29	12	41.4		
				Global *χ*_1_^2^=12.85, *P*=0.0003	
					
*Ethnicity*					
White	4787	1868	39.0	1	Reference
Other (non-white)	223	95	42.6	1.42	1.03–1.94
Missing	28	10	35.7		
				Global *χ*_1_^2^=4.68, *P*=0.0306	
					
*Marital status*					
Married/living as married	2824	840	29.8	1	Reference
Divorced/separated/widowed	667	289	43.3	1.97	1.62–2.40
Single	1494	826	55.3	1.29	1.09–1.53
Missing	53	18	34.0		
				Global *χ*_2_^2^=47.80, *P*<0.0001	
					
*Ever pregnant*^b^					
No	1589	855	53.8	1	Reference
Yes	3412	1104	32.4	0.75	0.63–0.89
Missing	37	14	37.8		
				Global *χ*_1_^2^=11.30, *P*=0.0008	
					
*Age at first pregnancy*^b^					
Never pregnant	1589	855	53.8	1	Reference
First pregnancy aged <20 years	1080	453	41.9	0.86	0.71–1.05
First pregnancy aged over 20 years	2303	640	27.8	0.67	0.55–0.80
Missing	66	25	37.9		
				Global *χ*_2_^2^=19.76, *P*=0.0001	
					
*Children*^b^					
No	2113	1096	51.9	1	Reference
Yes	2869	854	29.8	0.71	0.60–0.83
Missing	56	23	41.1		
				Global *χ*_1_^2^=17.06, *P*<0.0001	
					
*Number of children*b,^c^					
1	793	324	40.9	1	Reference
2	1170	288	24.6	0.75	0.59–0.95
3	563	148	26.3	0.89	0.67–1.19
4	190	49	25.8	0.82	0.54–1.24
5–8	86	25	29.1	1.11	0.64–1.94
Missing	67	20	29.9		
				Global *χ*_4_^2^=7.23, *P*=0.1242	
					
*Caesarean ever*b,^c^					
No	2367	704	29.7	1	Reference
Yes	481	145	30.2	1.12	0.88–1.43
Missing	21	5	23.8		
				Global *χ*_1_^2^=0.85, *P*=0.3574	
					
*Reproductive history*^d^					
Never pregnant	1589	855	53.8	1	Reference
First pregnancy <age 20 years, have children	867	335	38.6	0.79	0.64–0.99
First pregnancy <age 20 years, no children	205	113	55.1	1.01	0.73–1.40
First pregnancy ⩾age 20 years, have children	1981	513	25.9	0.61	0.50–0.75
First pregnancy ⩾age 20 years, no children	313	125	39.9	0.83	0.62–1.10
Missing	83	32	38.6		
				Global *χ*_4_^2^=25.55, *P*<0.0001	
					
*Current barrier contraception*					
No	4211	1611	38.3	1	Reference
Yes	811	354	43.7	1.05	0.87–1.27
Missing	16	8	50.0		
				Global *χ*_1_^2^=0.29, *P*=0.5907	
					
*Hormonal contraception*^e^					
Never pill user/no other current hormonal contraception	2416	649	26.9	1	Reference
Never pill user/currently use other hormonal contraception	200	101	50.5	1.58	1.13–2.21
Ex-pill user/no other current hormonal contraception	551	256	46.5	1.33	1.06–1.66
Ex-pill user/currently use other hormonal contraception	98	48	49.0	1.12	0.71–1.76
Current pill	1694	889	52.5	1.54	1.30–1.84
Missing	79	30	38.0		
				Global *χ*_4_^2^=27.33, *P*<0.0001	
					
*Physical activity*^f^					
Never	720	275	38.2	1	Reference
Ever	4239	1665	39.3	0.86	0.71–1.04
Missing	79	33	41.8		
				Global *χ*_1_^2^=2.40, *P*=0.1216	
					
*Smoking status*					
Never smoker	2340	862	36.8	1	Reference
Ex-smoker	851	264	31.0	0.88	0.73–1.07
Current smoker	1798	822	45.7	1.21	1.04–1.40
Missing	49	25	51.0		
				Global *χ*_2_^2^=11.83, *P*=0.0027	
					

Abbreviations: CI, confidence interval; hrHPV+ve, hrHPV positive; OR, odds ratio.

a Multivariate OR adjusted for age/smear, tertiary education/training, ethnicity, marital status, reproductive history, use of hormonal contraception and smoking status.

b Multivariate OR adjusted for age/smear, tertiary education/training, ethnicity, marital status, use of hormonal contraception and smoking status.

c Restricted to women who have had children.

d Ever been pregnant, age at first pregnancy and ever had children were all individually associated with hrHPV. As these variables are related, a composite variable was created and fitted in model.

e Women were classified into one of five categories on the basis of current use of oral contraceptive pill or other hormonal contraception, and on any previous oral contraceptive pill use.

f There was no effect on risk of hrHPV of physical activity (never/ever) or of frequency of physical activity (data not shown).
